# Supplementary material for: Genetic Variants Associated with the Age of Onset Identified by Whole-Exome Sequencing in Fatal Familial Insomnia
Source: Cells. 2023 Aug 12;12(16):2053. doi: 10.3390/cells12162053 (PMC10453322; doi:10.3390/cells12162053)
Supplement: Supplementary file 1 [file cells-12-02053-s001.zip › cells-2470520-supplementary.pdf]

Figure S1: Identified non-synonymous mutations in FFI patients at the marker level. Age of onset (top panel), presence (green) or absence (blue of marker (middle panel), and analyzed subjects (lower panel). Red and blue bars indicate female and male subjects (top panel).

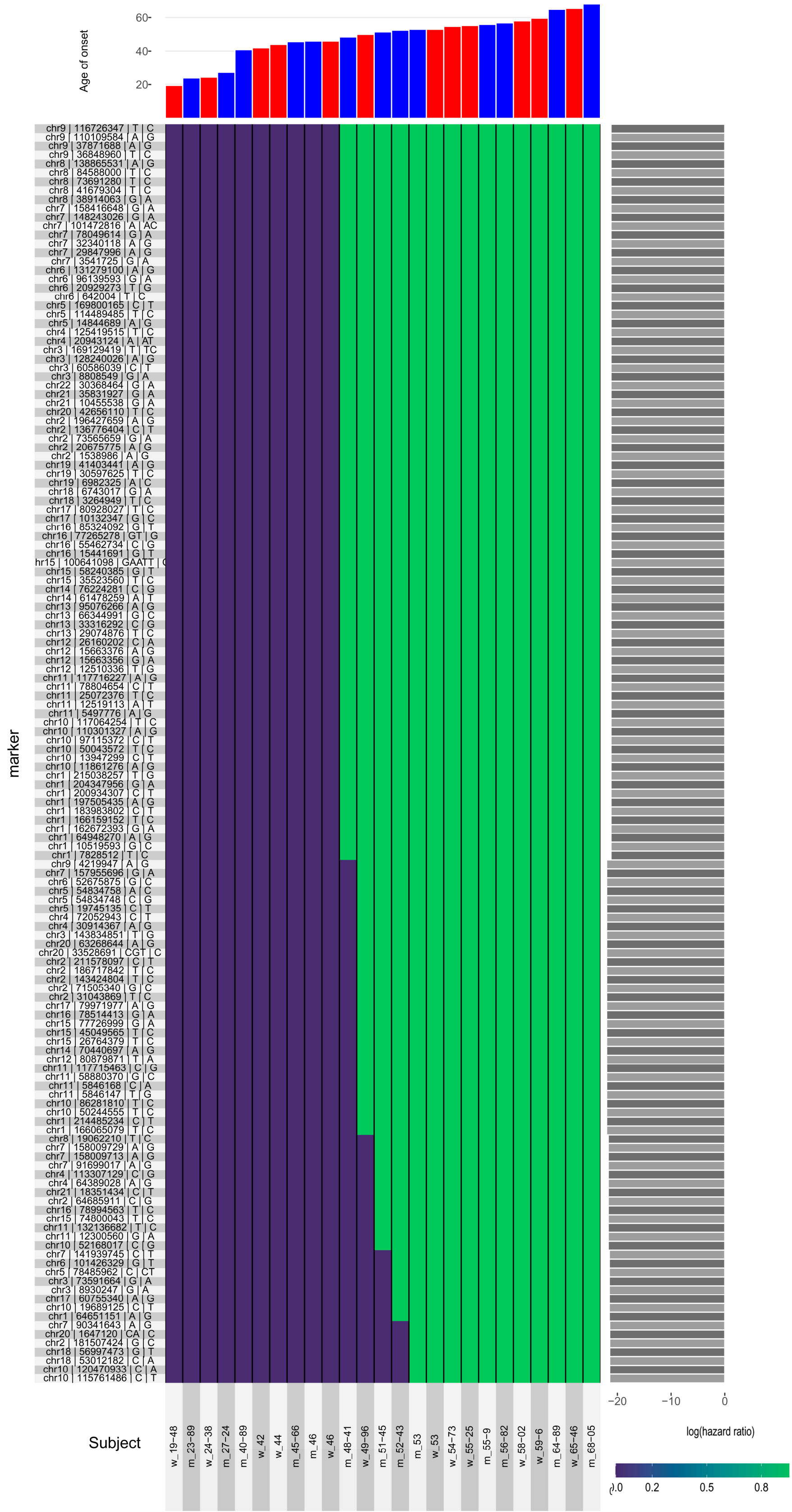

Table S1: Detailed information on the study cohort consisting of 25 samples of FFI patients; sex and age at onset are shown for each patient.

#	Diagnosis	Sex	Age at Onset
**1**	**FFI**	F	**19**
**2**		M	**23**
**3**		F	**24**
**4**		M	**27**
**5**		M	**40**
**6**		F	**42**
**7**		F	**44**
**8**		M	**45**
**9**		M	**46**
**10**		F	**46**
**11**		M	**48**
**12**		F	**49**
**13**		M	**51**
**14**		M	**52**
**15**		F	**53**
**16**		M	**53**
**17**		F	**54**
**18**		M	**55**
**19**		F	**55**
**20**		M	**56**
**21**		F	**58**
**22**		F	**59**
**23**		M	**64**
**24**		F	**65**
**25**		M	**68**

Table S2: Identification of most relevant genetic markers identified in FFI patients by WES according to their distribution pattern. CHR: chromosome; BP: base pairs; REF/ALT: reference/alternative allele; NMD: nonsense-mediated mRNA decay.

BP	CHR	SNP ID	Gene	REF	ALT	Location	Patient Age/Sex
116726347	9	rs10817927	*ASTN2*	T	C	Intronic (protein coding)	48m, 51m, 56m, 64m, 68m, 54w, 55w, 58w, 59w, 65w
110109584	9	rs579775	*PALM2AKAP2*	A	G		48m, 52m, 53m, 55m, 56m, 68m, 53w, 54w, 55w, 59w
37871688	9	rs776024	*AL138752.2*	A	G	Intronic (NMD)	48m, 51m, 52m, 53m, 55m, 56m, 64m, ,68m, 49w, 54w, 55w, 58w, 59w, 65w
36848960	9	rs10448236	*PAX5*	T	C		
138865531	8	rs11776837	*COL22A1*	A	G	Intronic (protein coding)	
84588000	8	rs1252206	*RALYL*	T	C		
73691280	8	rs7816524	*STAU2*	T	C		
41679304	8	rs1685324	*ANK1*	T	C	Intronic (NMD)	
38914063	8	rs7386365	*PLEKHA2*	G	A	Intronic (protein coding)	48m, 51m, 53m, 55m, 56m, 64m, 68m, 49w, 54w, 58w, 65w
